# Sex and gender differentials in the prevalence of obesity and its association with multimorbidity among reproductive-aged individuals in India

**DOI:** 10.3389/fpubh.2024.1496522

**Published:** 2025-01-07

**Authors:** Jogesh Murmu, Abhinav Sinha, Ritik Agrawal, Bhagyashree Rout, Srikanta Kanungo, Sanghamitra Pati

**Affiliations:** ^1^Department of Health Research, ICMR-Regional Medical Research Centre, Bhubaneswar, Odisha, India; ^2^South Asian Institute of Health Promotion, Bhubaneswar, Odisha, India

**Keywords:** reproductive-aging population, India, multimorbidity, chronic conditions, obesity, sex, gender, NFHS-5

## Abstract

**Introduction:**

The increase in the prevalence of obesity has become a common public health issue worldwide, with low- and middle-income countries (LMICs) like India witnessing an equal rise. It makes a considerable contribution to chronic diseases as it is a major risk factor for other chronic illnesses. Multimorbidity, or the presence of two or more chronic illnesses, is becoming more common in LMICs, resulting in poor health outcomes. However, research on obesity and multimorbidity in younger populations in LMICs is scarce, with most studies focusing on older persons. The study analyzed sex differences in the prevalence of obesity among reproductive-aged persons and its association with multimorbidity, as well as investigated their health-seeking behaviors.

**Methods:**

Data from the National Family Health Survey (NFHS-5) involving 751,831 females and 100,656 males were analyzed. Multimorbidity was defined by the presence of two or more chronic conditions out of the eight included chronic conditions. Multivariable logistic regression was applied to identify factors associated with obesity.

**Result:**

The prevalence of obesity was 48.90% (95% CI: 48.60–49.20%) among males and 57.10% (95% CI: 57.00–57.22%) among females. Waist-to-Hip Ratio (WHR) revealed higher obesity rates in females with multiple chronic conditions (70.8%) compared to males (65.1%). Males with multimorbidity had a 47% higher likelihood of having obesity (AOR: 1.47, 95% CI: 1.13–1.89, *p* < 0.003) compared to individuals without obesity.

**Conclusion:**

The study highlights high obesity prevalence among reproductive-aged individuals in India, with females having higher obesity rates overall. However, males with multimorbidity exhibit a significantly greater likelihood of obesity than males without. These findings emphasize the need for gender-specific public health strategies addressing obesity and multimorbidity, including promoting healthier diets, increasing physical activity, and improving disease management for both women and men.

## Introduction

Obesity has arisen as a major public health issue, with critical levels in many countries, including low- and middle-income countries (LMICs). It is a major risk factor for chronic illnesses such as cardiovascular disease, diabetes, musculoskeletal problems, and some malignancies (1). The World Health Organization (WHO) estimates that 890 million persons globally, representing 16% of the global adult population have obesity, including 18.3% of females and 13.7% of males (1). Waist-hip ratio (WHR) of ≥0.90 cm for males and ≥ 0.85 cm for females defined as obesity. WHR, measured as the ratio of waist circumference to hip circumference, is a commonly used body composition measurement that is a powerful predictor of obesity-related health consequences (2). Studies have shown that the WHR is a more accurate predictor for chronic diseases than body mass index (BMI), commonly used to measure obesity. This provides insight into body composition and contributes to chronic diseases due to their correlation with excess body fat (3, 4). A high WHR is closely linked to an increased risk of having obesity-related health diseases such as type 2 diabetes mellitus (T2DM), site-specific malignancies, cardiovascular events, chronic renal disease, musculoskeletal disorders, and infections (5, 6).

Multimorbidity, defined as the presence of two or more chronic illnesses in one person without focusing on a specific index disease, has become a norm in LMICs. It is increasingly associated with negative health outcomes such as disabilities, deaths, hospitalizations, lower quality of life, and increased use of healthcare services (7). Unhealthy lifestyle choices, such as insufficient physical exercise, poor food, smoking, and excessive alcohol consumption, have all contributed to the increasing prevalence of non-communicable diseases (NCDs), including obesity (8, 9).

Despite the significant healthcare expenses associated with multimorbidity and obesity, little is known about their prevalence in low- and middle-income countries (LMICs) (10). In India, obesity affects 40.3% of the population, with females having a greater prevalence (41.88%) than males (38.67%). Obesity rates are greater in cities (44.17%) than in rural areas (36.08%), and people over 40 had a higher prevalence (45.81%) than those under 40 (34.58%) (11). A Chinese study found a link between obesity and an increased risk of multimorbidity among people 65 and older, with the risk increasing with age (12).

Given the potential long-term impacts of early multimorbidity on both individuals and society, the reproductive-aged population is particularly vulnerable to the adverse effects of obesity. Studying sex differences in obesity and early onset of multimorbidity among Indian females of reproductive age provides valuable insights. Sex-specific analysis is crucial for understanding the progression of chronic conditions and implementing timely interventions. Such information is essential for shaping public health policies to reduce the incidence of these conditions and improve health outcomes. Thus, we examined sex differences in obesity prevalence and its correlates among reproductive-aged individuals (15–49 years for females, 15–54 years for males). Additionally, we explored the association between obesity and multimorbidity and investigated healthcare utilization among participants.

## Materials and methods

### Overview of data

In partnership with the Ministry of Health and Family Welfare, the International Institute for Population Sciences (IIPS) conducted the nationwide Family Health Survey (NFHS) in India, an extensively representative nationwide survey. The most recent survey, NFHS-5, collected information from 29 states and 7 Union Territories (UTs) between 2019 and 2021.

### Study design and study population

This study uses secondary data from the NFHS-5 dataset. The study included females aged 15–49 who were not pregnant and had not given birth in the 2 months before the survey, as well as males aged 15–54 years.

### Sample size and sampling technique

A two-stage selection procedure was employed to choose villages in rural areas and Census Enumeration Blocks (CEBs) in cities. Data were gathered using Computer-Assisted Personal Interviewing (CAPI) to ensure accuracy and efficiency. The sampling process used in NFHS-5 was a methodical approach to select households, assuring national and district-level representation. 751,831 females and 100,656 males of reproductive age took part in face-to-face interviews. The precise sample techniques and data collection methods utilized in NFHS-5 have already been published, providing methodological transparency and reproducibility (13).

### Data variable

#### Outcome characteristics

Anthropometric data, such as waist and hip circumferences, were obtained using Gulick Tape to act as a biomarker for obesity. These measures were used to compute the waist-to-hip ratio (WHR), which is a good indicator of body fat distribution and a predictor of abdominal obesity. Obesity was defined as a WHR of more than 0.9 for males and 0.85 for females.

#### Sociodemographic characteristics

The sociodemographic details collected from respondents encompassed age, sex, residence type (urban or rural), caste (classified into four categories), educational background (divided into four stages), employment status (employed or unemployed), wealth index (segmented into five quintiles), and current relationship status. The age for females was categorized as 15–19, 20–29, 30–39, and 40–49 years, while for males they were 15–19, 20–29, 30–39, 40–49, and 50–54 years. Marital status was categorized into “married” (currently married), “formerly/ever married” (including divorced, widowed, or separated), and “unmarried” (never married). Educational attainment was categorized as “no education” (no formal schooling), “up to primary” (less than 5 years of schooling), “up to secondary” (5 to 9 years of schooling), and “higher” (more than 10 years of schooling). Employment status was divided into “employed” (engaged in any form of occupation including professional, technical, managerial, clerical, sales, service, agricultural, skilled, or unskilled labor) and “unemployed” (not engaged in any work). The geographical region was classified into “north,” “central,” “east,” “northeast,” “west,” and “south.” Wealth categories included “poorest,” “poorer,” “middle,” “wealthier,” “richer,” and “richest,” based on household assets. Healthcare usage was categorized by facility type: “public,” “private,” “NGO/trust hospitals/clinics,” and “others” (such as pharmacies, home treatment, or alternative sources). Health insurance status was divided into “has a health insurance scheme” and “does not have a health insurance scheme. The parity of females was categorized into “nulliparous,” “primiparous,” and “multiparous.”

#### Multimorbidity

Multimorbidity was assessed through self-reported information on the presence of eight chronic conditions, including diabetes, hypertension, asthma, goiter or other thyroid disorders, cardiac disease, cancer, chronic kidney disease, and HIV. For the study, multimorbidity was classified into two groups: “absent” (having none or one chronic condition) and “present” (having two or more chronic conditions).

#### Statistical analysis

The statistical analysis was carried out using STATA 16 (StataCorp, College Station, Texas, USA). Data that were flagged, missing, or incomplete were removed before analysis, and variables were recoded as necessary. To overcome the differential probabilities of participant selection, NFHS sampling weights were used, ensuring the findings’ correctness and representativeness. The prevalence of chronic illnesses, obesity, and their predictors was calculated using weighted proportions, and the results were provided with 95% confidence intervals (CIs) to express uncertainty. All independent factors were subjected to unadjusted logistic regression, with findings presented as odds ratios (OR) and 95% confidence intervals. Following that, multivariable logistic regression was performed. The findings were presented as adjusted odds ratios (AOR) with 95% confidence intervals and significance determined by *p*-values less than 0.001.

#### Ethical consideration

This study posed no risk to participants, as it utilized secondary, anonymized data from the NFHS. Informed consent was obtained from all respondents during the original survey. Proper acknowledgment and citation of the dataset used in the analysis were ensured, following ethical guidelines.

## Results

### Sociodemographic characteristics

The average age of male respondents was 32.21 ± 11.21 years, ranging from 15 to 54 years, while that for the female respondents was 30.40 ± 9.88 years, ranging from 15 to 49 years. The majority of participants were between 20 and 29 years old. Around 75% of respondents lived in metropolitan areas, and more than half had completed secondary school. A significant 67.8% of females were unemployed, while 81.1% of males worked. The majority of individuals did not smoke tobacco. Furthermore, 63.4% of males and 67.9% of females did not have health insurance. Over half of the participants used public health services. Additional information is provided in Table 1.

**Table 1:** Table of sociodemographic characteristics across sexes.

Socio-demographic characteristics	Female (*N* = 7,15,831)		Socio-demographic characteristics	Male (*N* = 1,00,656)	
	Unweighted *n* (%)*	Weighted *n* (%)*		Unweighted *n* (%)*	Weighted *n* (%)*
Age group			Age group		
15–19 years	1,21,042 (16.91)	1,21,173 (16.93)	15–19 years	16,485 (16.38)	16,210 (16.10)
20–29 years	2,34,041 (32.70)	2,33,465 (32.61)	20–29 years	28,395 (28.21)	28,178 (27.99)
30–39 years	1,97,008 (27.52)	1,95,845 (27.36)	30–39 years	25,852 (25.68)	25,867 (25.70)
40–49 years	1,63,740 (22.87)	1,65,348 (23.10)	40–49 years	21,440 (21.30)	21,804 (21.66)
			50–54 years	8,484 (8.43)	8,596 (5.54)
Residence			Residence		
Rural	1,76,187 (24.61)	2,30,518 (32.20)	Rural	25,941 (25.77)	35,166 (34.94)
Urban	5,39,644 (75.39)	4,85,313 (67.80)	Urban	74,715 (74.23)	65,490 (65.06)
Education			Education		
No education	1,65,631 (23.14)	1,61,025 (22.49)	No education	12,138 (12.06)	11,951 (11.87)
Primary	84,163 (11.76)	84,195 (11.76)	Primary	11,572 (11.50)	12,103 (12.02)
Secondary	3,66,079 (51.14)	3,59,505 (50.22)	Secondary	59,370 (58.98)	57,302 (56.93)
Higher	99,958 (13.96)	1,11,106 (15.52)	Higher	17,576 (17.46)	19,300 (19.17)
Marital status			Marital status		
Unmarried	1,78,777 (24.97)	1,69,459 (23.67)	Unmarried	36,380 (36.14)	36,087 (35.85)
Married	5,06,939 (70.82)	5,15,980 (72.08)	Married	62,723 (62.31)	63,141 (62.73)
Formerly/ever married	30,115 (4.21)	30,392 (4.25)	Formerly/ever married	1,553 (1.54)	1,428 (1.42)
Occupation (*N* = 1,07,414)^†^			Occupation (*N* = 1,00,418)^†^		
Unemployed	72,859 (67.83)	74,653 (69.50)	Unemployed	18,949 (18.87)	18,044 (17.97)
Employed	34,555 (32.17)	32,761 (30.50)	Employed	81,469 (81.13)	82,374 (82.03)
Caste (*N* = 6,81,539)^†^			Caste (*N* = 96,146)^†^		
Scheduled caste	1,38,363 (20.30)	1,56,998 (23.04)	Scheduled caste	19,021 (19.78)	21,052 (21.90)
Scheduled tribe	1,34,349 (19.71)	66,794 (9.80)	Scheduled tribe	19,215 (19.99)	9,356 (9.73)
OBC	2,73,530 (40.13)	3,07,136 (45.07)	OBC	38,831 (40.39)	43,424 (45.16)
Others	1,35,297 (19.85)	1,50,611 (22.10)	Others	19,079 (19.84)	22,341 (23.21)
Alcohol consumption			Alcohol consumption		
No	7,02,939 (98.12)	7,10,475 (99.25)	No	74,436 (73.95)	77,575 (77.07)
Yes	13,438 (1.88)	5,355 (0.75)	Yes	26,220 (26.05)	23,081 (22.93)
Tobacco use			Tobacco use		
No	6,69,076 (93.47)	6,85,863 (95.81)	No	56,835 (56.46)	59,476 (59.09)
Yes	46,755 (6.53)	29,968 (4.19)	Yes	43,821 (43.54)	41,180 (40.91)
Wealth index			Wealth index		
Poorest	1,48,649 (20.77)	1,33,130 (18.60)	Poorest	19,640 (19.51)	16,874 (16.76)
Poorer	1,59,059 (22.22)	1,43,951 (20.11)	Poorer	22,412 (22.27)	19,923 (19.79)
Middle	1,50,128 (20.97)	1,47,444 (20.60)	Middle	21,500 (21.36)	21,468 (21.33)
Richer	1,37,885 (19.26)	1,48,832 (20.79)	Richer	19,930 (19.80)	22,373 (22.23)
Richest	1,20,110 (16.78)	1,42,475 (19.90)	Richest	17,174 (17.06)	20,017 (19.89)
Health insurance			Health insurance		
No	4,86,082 (67.90)	5,02,150 (70.15)	No	63,849 (63.43)	66,510 (66.08)
Yes	2,29,749 (32.10)	2,13,681 (29.85)	Yes	36,807 (36.57)	34,146 (33.92)
Treatment facility (*N* = 2,57,885)^†^			Treatment facility (*N* = 28,541)^†^		
Public	1,69,395 (65.69)	1,51,824 (58.87)	Public	18,080 (63.35)	15,808 (55.39)
Private	86,151 (33.41)	1,03,363 (40.08)	Private	10,207 (35.76)	12,381 (43.38)
NGO/Trust	735 (0.29)	948 (0.37)	NGO/Trust	102 (0.36)	139 (0.49)
Others	1,604 (0.62)	1,750 (0.68)	Others	152 (0.53)	212 (0.74)
Parity			Parity		
Nulliparous	2,226,762 (31.68)	2,19,617 (30.68)	Nulliparous	-	-
Primiparous	98,155 (13.71)	1,01,739 (14.21)	Primiparous	-	-
Multiparous	3,90,914 (54.61)	3,94,475 (55.11)	Multiparous	-	-

OBC, Other backward class; NGO, Non-government organization.

*Column percentage, ^†^missing, and no information participants were removed.

### Profile of various chronic conditions

The prevalence of multimorbidity was 2.72% in males and 2.14% in females. HIV emerged as the most common chronic disease, affecting 21.18% of females (95% CI: 20.09–21.42) and 9.53% of males (95% CI: 9.35–9.71). Hypertension was the second most prevalent condition, affecting 4.76% of females (95% CI: 4.71–4.81) and 3.30% of males (95% CI: 3.18–3.41). Cancer had the lowest prevalence, affecting 0.12% of females (95% CI: 0.11–0.13) and 0.19% of males (95% CI: 0.16–0.22). Table 2 provides a full summary of chronic illnesses.

**Table 2:** Profile of chronic conditions across sexes.

Female		Male	
Chronic conditions	Prevalence *n*, % *(95% CI)	Chronic conditions	Prevalence *n*, % *(95% CI)
Diabetes (*n* = 7,07,647)	13,426, 1.90 (1.86–1.93)	Diabetes (*n* = 99,542)	2,728, 2.74 (2.64–2.84)
Hypertension (*n* = 7,10,213)	33,826, 4.76 (4.71–4.81)	Hypertension (*n* = 99,838)	3,292, 3.30 (3.18–3.41)
Asthma (*n* = 7,13,091)	11,586, 1.62 (1.59–1.65)	Asthma (*n* = 1,00,205)	1,385, 1.38 (1.31–1.45)
Thyroid (*n* = 7,10,942)	19,204, 2.70 (2.66–2.74)	Thyroid (*n* = 1,00,195)	524, 0.52 (0.47–0.57)
Cardiac disease (*n* = 7,12,294)	5,112, 0.72 (0.69–0.74)	Cardiac disease (*n* = 1,00,177)	1,045, 1.04 (0.98–1.11)
Cancer (*n* = 7,12,298)	876, 0.12 (0.11–0.13)	Cancer (*n* = 1,00,167)	189, 0.19 (0.16–0.22)
Chronic kidney disease (*n* = 7,11,870)	4,128, 0.58 (0.56–0.59)	Chronic kidney disease (*n* = 1,00,179)	986, 0.98 (0.92–1.05)
HIV (*n* = 1,07,542)	22,774, 21.18 (20.09–21.42)	HIV (*n* = 1,00,656)	9,591, 9.53 (9.35–9.71)
Multimorbidity (*n* = 7,15,831)	15,284, 2.14 (2.10–2.17)	Multimorbidity (*n* = 1,00,656)	2,735, 2.72 (2.61–2.82)

CI, Confidence interval; HIV, Human immunodeficiency virus.

*Column percentage.

### Prevalence of obesity with sociodemographic characteristics

**Figure 1:**
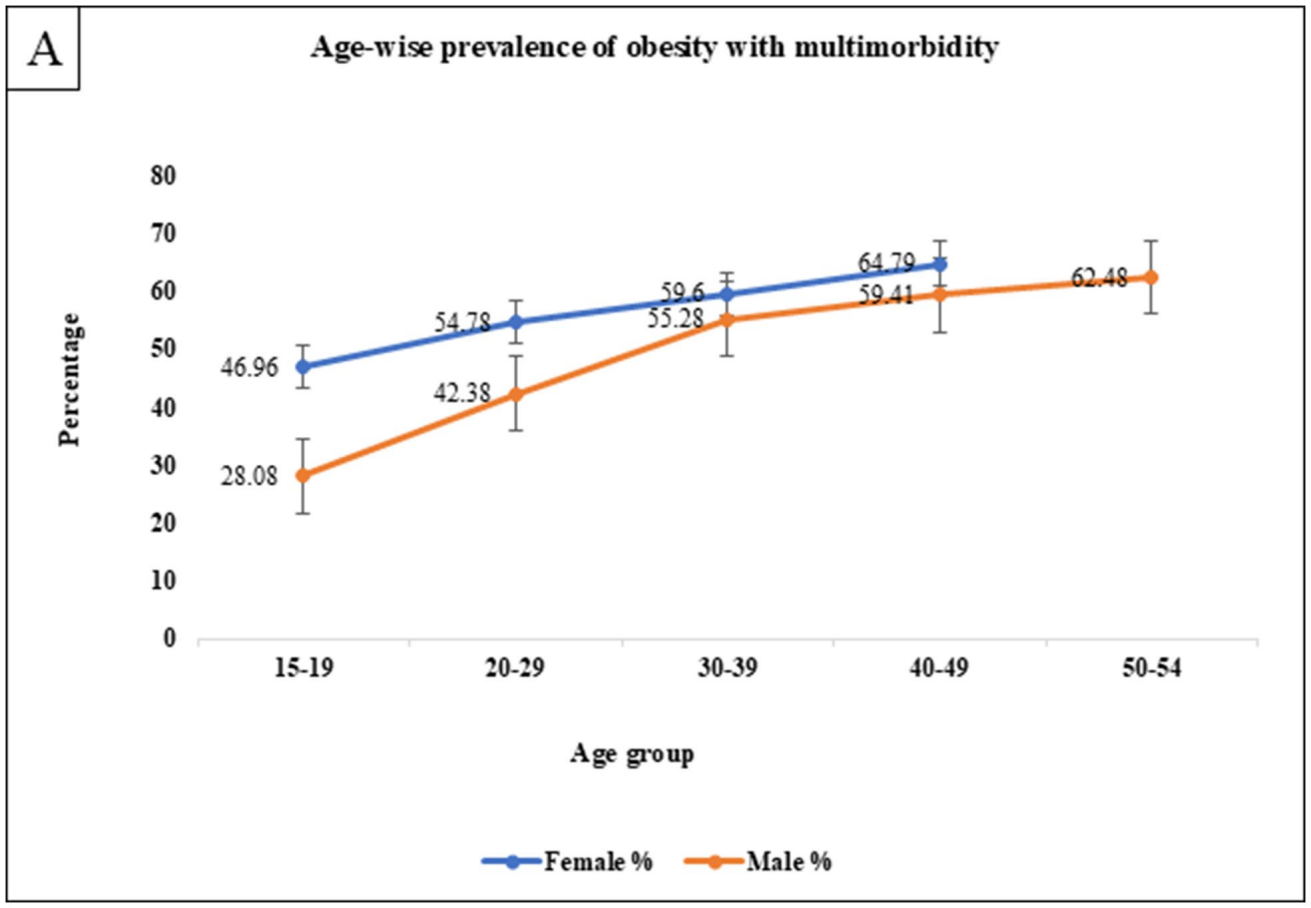
Age-wise sex differential prevalence of obesity with multimorbidity.

The overall prevalence of obesity was 48.89% (95% CI: 48.57–49.20) among males and 57.11% (95% CI: 57.00–57.23) among females. As detailed in Table 3, obesity rates increased with age, peaking at 62.48% for males aged 50 to 54 years and 64.79% for females aged 40 to 49 years. Obesity was more prevalent among rural residents and women without formal education (59.31%). Higher rates were observed among unemployed women (57.83%) and employed men (52.10%). Alcohol consumption was strongly associated with obesity, with 61.05% of males and 54.07% of females affected. Additionally, individuals in the richest wealth quintile exhibited higher obesity rates, with 55.42% of males and 60.96% of females classified as people having obesity. Multimorbidity further amplified the prevalence of obesity, affecting 65.12% of males and 70.78% of females, indicating a slight predominance among females. Nulliparous women constituted a smaller proportion (50.29%, *n* = 1,06,539) compared to both primiparous (61.61%, *n* = 61,266) and multiparous (59.69%, *n* = 2,31,500). The predominance of multiparous women underscores the potential influence of parity on obesity and multimorbidity outcomes (Figure 1).

**Table 3:** Prevalence of obesity with sociodemographic characteristics.

Sociodemographic characteristics	Female (*N* = 6,99,155)		Male (*N* = 96,010)	
	*N*	% *(95% CI)	*N*	% *(95% CI)
Age group			Age group	
15–19 years	55,057	46.96 (46.67–47.24)	4,304	28.08 (27.36–28.79)
20–29 years	1,24,917	54.78 (54.57–54.98)	11,283	42.38 (41.78–42.97)
30–39 years	1,14,276	59.60 (59.37–59.98)	13,680	55.28 (54.66–55.90)
40–49 years	1,05,055	64.79 (64.56–65.02)	12,438	59.41 (58.74–60.07)
50-54 years			5,231	62.48 (61.43–63.52)
Residence			Residence	
Rural	1,33,100	60.24 (60.03–60.44)	16,811	51.69 (51.14–52.23)
Urban	2,66,205	55.67 (55.52–55.81)	30,126	47.45 (47.06–47.84)
Education			Education	
No education	93,918	59.31 (59.06–59.55)	5,850	50.76 (49.84–51.67)
Primary	48,362	58.25 (57.91–58.58)	6,061	51.80 (50.88–52.70)
Secondary	1,95,591	55.65 (55.48–55.81)	25,929	47.24 (46.82–47.66)
Higher	61,434	57.81 (57.51–58.11)	9,097	50.83 (50.09–51.56)
Marital status			Marital status	
Unmarried	78,104	47.91 (47.66–48.15)	12,067	35.55 (35.04–36.06)
Married	3,03,445	59.93 (59.80–60.07)	34,191	56.36 (55.96–56.75)
Formerly/ever married	17,756	59.54 (58.98–60.1)	679	48.62 (45.98–51.29)
Occupation (*N* = 1,04,957)^†^			Occupation (*N* = 95,784)^†^	
Unemployed	42,113	57.83 (57.46–58.18)	5,767	33.96 (33.24–34.67)
Employed	17,198	53.52 (52.97–54.06)	41,053	52.10 (51.74–52.44)
Caste (*N* = 6,65,577)^†^			Caste (*N* = 91,719)^†^	
Scheduled caste	88,201	57.30 (57.05–57.55)	9,847	48.85 (48.15–49.54)
Scheduled tribe	35,308	53.64 (53.25–54.01)	3,692	40.58 (39.56–41.59)
OBC	1,65,086	55 (54.81–55.17)	20,094	48.54 (48.05–49.02)
Others	87,852	60.32 (60.06–60.57)	11,010	52.28 (51.60–52.95)
Alcohol consumption			Alcohol consumption	
No	3,96,063	57.08 (56.96–57.19)	34,979	47.34 (46.97–47.70)
Yes	3,242	61.05 (59.72–62.36)	11,958	54.07 (53.41–54.72)
Tobacco use			Tobacco use	
No	3,82,698	57.16 (57.04–57.27)	26,379	46.91 (46.49–47.32)
Yes	16,607	56.06 (55.49–56.63)	20,558	51.69 (51.19–52.18)
Wealth index			Wealth index	
Poorest	73,530	56.08 (55.81–56.35)	7,230	44.31 (43.55–45.08)
Poorer	78,591	55.35 (55.09–55.61)	8,726	45.40 (44.70–46.11)
Middle	80,917	55.75 (55.49–56.01)	9,790	47.30 (46.62–47.98)
Richer	83,769	57.54 (57.28–57.79)	10,953	51.41 (50.73–52.08)
Richest	82,497	60.96 (60.70–61.22)	10,237	55.42 (54.69–56.13)
Multimorbidity			Multimorbidity	
No	3,88,610	56.81 (56.69–56.93)	45,187	48.42 (48.09–48.74)
Yes	10,695	70.78 (70.05–71.50)	1,750	65.12 (63.29–66.93)
Health insurance			Health insurance	
No	2,79,643	57.19 (57.05–57.33)	30,386	48.24 (47.85–48.63)
Yes	1,19,662	56.94 (56.72–57.15)	16,551	50.12 (49.58–50.66)
Treatment facility (*N* = 2,53,009)^†^			Treatment facility (*N* = 27,438)^†^	
Public	90,627	60.59 (60.34–60.84)	7,614	49.94 (49.14–50.73)
Private	58,073	57.62 (57.31–57.92)	6,028	52.35 (51.45–53.25)
NGO/Trust	519	56.20 (52.96–59.46)	83	61.67 (52.72–69.72)
Others	1,028	59.40 (57.03–61.71)	84	42.19 (35.26–49.40)
Parity			Parity	
Nulliparous	1,06,539	50.29 (50.07–50.49)	-	-
Primiparous	61,266	61.61 (61.30–61.91)	-	-
Multiparous	2,31,500	59.69 (59.53–59.84)	-	-

CI, Confidence interval; OBC, Other backward class; NGO, Non-government organization.

*Row percentage.

^†Population characteristics of the respective variables differ from those of the exposure group.^

### Association of obesity with multimorbidity

A univariate logistic regression analysis revealed significant associations of obesity with various factors, including age, marital status, residence, occupation, caste, and wealth index, in both men and women. Married women were 1.70 times more likely to have obesity [OR: 1.70, (95% CI: 1.48–1.95)] than unmarried women, while married men had 1.54 times greater chances [OR: 1.70, (95% CI: 1.32–1.81)] of having obesity than their unmarried counterparts. Furthermore, unemployment was linked to a 2.11-fold higher incidence of obesity in women [OR: 2.11, (95% CI: 1.99–2.25)].

Women aged 40 to 49 had 1.95 times higher risks of having obesity [AOR: 1.95, (95% CI: 1.68–2.28), *p* < 0.001] than those aged 15 to 19. Men aged 50–54 had 3.27 times greater risks [AOR: 3.27, (95% CI: 2.52–4.25), *p* < 0.001]. Women living in urban areas were 15% more likely to have obesity [AOR: 1.15, (95% CI: 1.07–1.25), *p* < 0.001] than those living in rural areas.

Marriage was found to be a significant predictor of obesity, with married women having 1.70 times higher chances [AOR: 1.70, (95% CI: 1.48–1.95), *p* < 0.001] and married men displaying 1.54 times larger odds [AOR: 1.54, (95% CI: 1.32–1.81), *p* < 0.001] than their unmarried counterparts. Men with the highest wealth index were 1.44 times more prone to have obesity [AOR: 1.44, (95% CI: 1.22–1.72)].

Men with multimorbidity were 47% more likely to have obesity [AOR: 1.47, (95% CI: 1.13–1.89), *p* < 0.003] than those without it. In contrast, tobacco use exhibited a negative connection with obesity, as male tobacco users were 9% less likely to have obesity compared to non-users [AOR: 0.91, (95% CI: 0.82–1.00), *p* = 0.052]. Multiparous women had 16% lower odds of having obesity [AOR: 0.84, (95% CI: 0.77–0.92), *p* < 0.001] compared to both nulliparous and primiparous women (Table 4).

**Table 4:** Association of obesity with sociodemographic characteristics using univariable and multivariable regression.

Socio-demographic characteristics	Female (*N* = 6,99,155)			Male (*N* = 96,010)		
	Univariable OR, 95% CI	Multivariable AOR, 95% CI	*p*-value**	Univariable OR, 95% CI	Multivariable AOR, 95% CI	*p*-value**
Age group				Age group		
15–19 years	Reference			Reference		
20–29 years	1.37 (1.34–1.39)	1.22 (1.08–1.38)	0.001	1.88 (1.75–2.03)	1.54 (1.26–1.88)	<0.001
30–39 years	1.66 (1.63–1.70)	1.52 (1.32–1.75)	<0.001	3.17 (2.94–3.41)	2.31 (1.83–2.92)	<0.001
40–49 years	2.08 (2.03–2.12)	1.95 (1.68–2.28)	<0.001	3.75 (3.47–4.05)	2.57 (2.03–3.27)	<0.001
50–54 years	-	-		4.26 (3.87–4.69)	3.27 (2.52–4.25)	<0.001
Residence				Residence		
Urban	1.21 (1.18–1.23)	1.15 (1.07–1.25)	<0.001	1.18 (1.22–1.25)	1.00 (0.89–1.12)	0.992
Rural	Reference			Reference		
Marital status				Marital status		
Unmarried	Reference			Reference		
Married	1.62 (1.60–1.65)	1.70 (1.48–1.95)	<0.001	2.34 (2.22–2.46)	1.54 (1.32–1.81)	<0.001
Formerly/ever married	1.60 (1.54–1.66)	1.54 (1.26–1.88)	<0.001	1.71 (1.42–2.07)	1.16 (0.77–1.75)	0.459
Education				Education		
No education	1.16 (1.14–1.18)	0.97 (0.89–1.05)	0.455	1.15 (1.07–1.23)	0.87 (0.76–1.01)	0.072
Primary	1.11 (1.09–1.14)	0.97 (0.88–1.06)	0.516	1.20 (1.12–1.29)	1.01 (0.87–1.16)	0.882
Secondary	Reference			Reference		
Higher	1.09 (1.07–1.12)	1.04 (0.95–1.14)	0.405	1.15 (1.08–1.23)	1.10 (0.97–1.25)	0.122
Occupation (*N* = 1,04,957)^†^				Occupation (*N* = 95,784)^†^		
Unemployed	Reference			Reference		
Employed	0.84 (0.81–0.87)	0.73 (0.69–0.78)	<0.001	2.11 (1.99–2.25)	1.14 (0.96–1.36)	0.122
Caste (*N* = 6,65,577)^†^				Caste (*N* = 91,719)^†^		
Scheduled caste	1.16 (1.13–1.18)	1.11 (1.00–1.24)	0.047	1.39 (1.29–1.52)	1.38 (1.18–1.62)	<0.001
Scheduled tribe	Reference			Reference		
OBC	1.05 (1.03–1.08)	1.01 (0.91–1.11)	0.846	1.38 (1.28–1.48)	1.20 (1.04–1.38)	0.011
Others	1.31 (1.28–1.35)	1.31 (1.17–1.48)	<0.001	1.61 (1.47–1.74)	1.19 (1.01–1.40)	0.037
Alcohol				Alcohol		
No	Reference			Reference		
Yes	1.18 (1.11–1.25)	1.13 (0.88–1.44)	0.319	1.31 (1.24–1.38)	1.11 (0.99–1.23)	0.050
Tobacco				Tobacco		
No	Reference			Reference		
Yes	0.96 (0.93–0.98)	0.96 (0.84–1.09)	0.581	1.21 (1.15–1.27)	0.91 (0.82–1.00)	0.052
Wealth index				Wealth index		
Poorest	1.03 (1.01–1.05)	1.09 (1.00–1.20)	0.050	0.96 (0.89–1.02)	0.96 (0.84–1.11)	0.628
Poorer	Reference			Reference		
Middle	1.02 (0.99–1.04)	1.00 (0.92–1.20)	0.937	1.07 (1.01–1.15)	1.04 (0.92–1.18)	0.532
Richer	1.09 (1.07–1.11)	0.94 (0.86–1.03)	0.212	1.27 (1.18–1.36)	1.25 (1.09–1.44)	0.001
Richest	1.26 (1.23–1.28)	1.10 (0.98–1.22)	0.089	1.49 (1.38–1.62)	1.44 (1.22–1.72)	<0.001
Multimorbidity				Multimorbidity		
No	Reference			Reference		
Yes	1.84 (1.75–1.94)	1.09 (0.94–1.26)	0.235	1.99 (1.67–2.36)	1.47 (1.13–1.89)	0.003
Health insurance				Health insurance		
No	Reference			Reference		
Yes	0.99 (0.97–1.00)	0.98 (0.92–1.04)	0.535	1.08 (1.03–1.13)	1.00 (0.92–1.10)	0.854
Treatment facility (*N* = 2,53,009)^†^				Treatment facility (*N* = 27,438)^†^		
Public	1.05 (0.92–1.20)	1.10 (0.77–1.57)	0.595	1.36 (0.83–2.25)	1.16 (0.71–1.91)	0.542
Private	0.93 (0.81–1.06)	0.92 (0.64–1.32)	0.667	1.51 (0.91–2.48)	1.33 (0.81–2.19)	0.257
NGO/Trust	0.87 (0.68–1.12)	0.94 (0.50–1.74)	0.846	2.20 (1.05–4.64)	1.65 (0.77–3.49)	0.191
Others	Reference			Reference		
Parity				Parity		
Nulliparous	Reference			-	-	
Primiparous	0.63 (0.61–0.64)	1.10 (0.97–1.26)	0.127	-	-	
Multiparous	0.92 (0.90–0.94)	0.84 (0.77–0.92)	<0.001	-	-	

OR, Odds ratio; AOR, Adjusted odds ratio; CI, Confidence interval; OBC, Other backward class; NGO, Non-government organization.

**p-value<0.001. ^†^Population characteristics of the respective variables differ from those of the exposure group.

## Discussion

Our analysis reveals that obesity affects 48.89% of men (95% CI: 48.57–49.20) and 57.11% of women (95% CI: 57.00–57.23) in India. Significant risk factors for obesity identified include age, with the highest prevalence observed among women aged 40–49 and men aged 50–54; urban residency for women; the wealthiest quintile for men; marital status; and multimorbidity in men. Hypertension and HIV were identified as the most common chronic condition followed by diabetes and asthma across both genders.

The observed gender disparity in obesity aligns with earlier studies. Sinha et al. found that obesity was more prevalent among women (54.20%) compared to men (45.80%) (14), a trend also reported by Ruopeng An and Wang et al. (15–17). This difference may be attributed to the combination of hormonal, genetic, lifestyle, and cultural factors. For instance, estrogen promotes fat storage in women (18), while genetic predispositions may increase their vulnerability to obesity (19). Additionally, gendered lifestyle factors such as poor dietary habits, physical inactivity, and stress are known contributors to elevated obesity rates among women (20, 21). Health disparity has been similarly observed in LMICs, as evidenced by a study in Nepal and among tribal older adults reporting that one-fourth of the population experienced multimorbidity, highlighting the challenges of health conditions in these settings (22, 23). The escalating obesity rates in India can be linked to rapid urbanization, increased mechanized transport, consumption of processed foods, sedentary behaviors, and diets high in calories but low in nutrients (24).

Age also emerged as a critical factor in obesity, with older age being a recognized risk for having obesity and related non-communicable diseases (15). Venkatrao et al. reported that obesity rates were notably higher among individuals over 40 years (45.81%) compared to those under 40 years (34.58%) (11). This underscores the importance of addressing both immutable factors like age and biological sex, as well as modifiable factors such as educational attainment and physical activity, in obesity prevention strategies.

Alcohol consumption was significantly associated with obesity, with 61.05% of women and 54.07% of men affected. This aligns with evidence linking alcohol’s high caloric content and metabolic effects to weight gain, particularly in women (25, 26). Conversely, tobacco use was inversely related to obesity among men, with 51.69% of men having obesity reporting tobacco use. This aligns with previous studies indicating that smoking may suppress appetite and increase energy expenditure, thereby contributing to lower body weight (27). However, the health risks associated with tobacco use complicate its role in obesity management, suggesting a need for comprehensive public health strategies that address both tobacco cessation and obesity prevention (28, 29).

The study also highlights a positive association between wealth, urban residence, and obesity in reproductive-aged women. Higher obesity rates among wealthier individuals are likely influenced by dietary changes, reduced physical activity associated with urban living, and other related factors (30, 31). The increasing prevalence of abdominal obesity can be linked to the adoption of sedentary lifestyles and Western dietary patterns high in sugars, fats, and preservatives (32–34). Our findings demonstrate that sociodemographic factors such as education and employment contribute to sex-specific obesity trends. For women, the protective effect of employment likely reflects increased physical activity in certain occupational roles. Conversely, higher education does not confer substantial protection against obesity, likely due to behavioral and cultural influences. These findings align with studies by Anekwe et al. and Wang D et al., emphasizing the complex, multifactorial nature of obesity determinants (35, 36). Parity significantly influences reproductive health outcomes, particularly weight retention and obesity risk among women. The physiological changes during pregnancy and the postpartum period often result in weight gain, which can persist and become more pronounced with multiple pregnancies. Makama et al. report that 20% of women experience postpartum weight retention, emphasizing its public health relevance and the need for sex-sensitive interventions (37).

Our study confirms a strong association between obesity and multimorbidity, especially in men. Kivimaki et al. showed that a higher BMI markedly raises the risk of developing various cardiovascular and metabolic conditions, with overweight individuals being twice as likely and those who are severely obese more than 10 times as likely to encounter these conditions compared to those with a normal BMI (38). Obesity is a major contributor to various health issues, with recent research suggesting that people with obesity tend to experience multimorbidity earlier than those of normal weight (39–42).

### Policy implication

The observed connection between obesity and multimorbidity in both sexes underscores the necessity for sex- and gender-sensitive policies and strategies to address this issue effectively. To reduce the risk of multimorbidity, it is essential to implement early interventions and preventive measures, particularly in regions with limited healthcare resources. Strategies could include integrating nutrition education into public health campaigns, implementing stricter food labeling regulations, promoting access to healthy food in workplaces, and designing culturally relevant educational programs to encourage healthier lifestyles (43, 44). Public health policies must also account for family-level interventions, as multimorbidity tends to cluster in urban, affluent populations, increasing healthcare expenditures (45). Integrating NCD risk reduction into medical training and fostering preventive practices in clinical settings can prepare healthcare professionals to tackle these challenges more effectively (46). A comprehensive approach that incorporates sex- and gender-sensitive and family-centered strategies is essential to mitigate the burden of obesity and multimorbidity among reproductive-age groups in India. Policymakers should prioritize improving healthcare access and affordability while promoting physical activity. This study underscores the importance of a holistic, gender-focused framework for addressing obesity and multimorbidity, particularly in India and other low- and middle-income countries (LMICs).

### Strength and limitation

A major strength of this study is its use of anthropometric measurements, such as hip and waist circumference, to assess obesity, providing accurate indications of the disease. Furthermore, using data from a nationally representative survey increases the applicability of the findings. However, the cross-sectional nature of the data limits the ability to identify causal links. Furthermore, relying on self-reported chronic diseases may add bias, reducing the precision of prevalence estimates.

## Conclusion

This study reveals a significant sex and gender disparity in obesity and multimorbidity among India’s reproductive-aged population, with females disproportionately affected by obesity. These findings call for sex- and gender-specific public health strategies to address the rising burden of NCDs. Targeted interventions promoting healthier diets, increased physical activity, and lifestyle changes are crucial for women. Policies must address root causes such as sedentary behavior and poor nutrition. The higher prevalence of obesity among females underscores the need for targeted interventions addressing female-specific factors such as hormonal influences, sociocultural norms, and lifestyle patterns. The stronger association between multimorbidity and obesity in males highlights that males with existing chronic conditions may require more tailored strategies for weight management and prevention of further complications. Strengthening healthcare systems to ensure equitable, accessible care, particularly for women from disadvantaged backgrounds, is essential. Integrating prevention with better healthcare delivery will improve health outcomes and reduce the burden on the healthcare system.

## Data Availability

Publicly available datasets were analyzed in this study. This data can be found at: International Institute for Population Sciences (IIPS) - https://www.iipsindia.ac.in/.
